# First Reported Case of Hemoglobin Graz in the United States: Implications for Misleading Hemoglobin A_1c_ Results

**DOI:** 10.1210/jcemcr/luae242

**Published:** 2024-12-26

**Authors:** Luke Miller, Yaniv Maddahi, Matthew Shelly, Sudip Nanda, Mohammad Ishaq Arastu

**Affiliations:** Lewis Katz School of Medicine, Temple University, Philadelphia, PA 19140, USA; Lewis Katz School of Medicine, Temple University, Philadelphia, PA 19140, USA; Lewis Katz School of Medicine, Temple University, Philadelphia, PA 19140, USA; Department of Cardiology, St. Luke's University Health Network, Bethlehem, PA 18015, USA; Department of Endocrinology, St. Luke's University Health Network, Bethlehem, PA 18015, USA

**Keywords:** HbA_1c_, hemoglobin, Graz, hemoglobinopathy

## Abstract

Routine serum studies in a female patient with sustained prediabetic glycated hemoglobin A_1c_ (HbA_1c_) levels, controlled on metformin, yielded an unexpected finding: an elevated HbA_1c_ value of ≥14.9% (≥139 mmol/mol) (normal reference range, <5.7% to <39 mmol/mol). Estimated average glucose (EAG) (normal reference range, <126 mg/dL to <7 mmol/L) is a linearly corresponding blood glucose value calculated from HbA_1c_ measurements that reflects the average glycemic status over the preceding 3 months. Caution must be used when the EAG provided by the HbA_1c_ does not align with blood glucose values obtained around the same period. Our patient carries a rare heterozygous pathogenic variant affecting the β subunit called hemoglobin Graz (Hb Graz), characterized by a histidine for leucine substitution, resulting in clinically silent Hb abnormalities. Individuals without diabetes carrying the Hb Graz pathogenic variant exhibit significantly higher HbA_1c_ values when analyzed by high-performance liquid chromatography. Alternative methods of quantifying glycemic control are suggested if the possibility of a confounding variable exists, such as when a HbA_1c_-blood glucose mismatch occurs or unexplainable HbA_1c_ levels are detected.

## Introduction

Elevated glycated hemoglobin A_1c_ (HbA_1c_) levels generally indicate increased glucose binding to hemoglobin (Hb), implying hyperglycemia and risk for diabetes. Normal HbA_1c_ levels are defined as less than 5.7% (<39 mmol/mol); prediabetes between 5.7% and 6.4% (39 to 46 mmol/mol); and diabetes greater than or equal to 6.5% (≥48 mmol/mol) [1]. Institutions use different methods for measuring HbA_1c_, all of which may be affected by hemoglobinopathies [2]. Individuals without diabetes carrying the Hb Graz pathogenic variant exhibit HbA_1c_ values of 47.4% to 50.4% (495-527 mmol/mol) when analyzed by high-performance liquid chromatography (HPLC) [3]. Being a silent hemoglobinopathy, Hb Graz can unknowingly affect clinical treatment for patients. HbA_1c_ is crucial in determining patient treatment, including the need for a pharmacologic regimen, diet/lifestyle changes, or a combination of both. Having an erroneous HbA_1c_ level can affect patient safety, and for this reason, alternative methods of quantifying HbA_1c_ are suggested if there is the possibility of a hemoglobinopathy or a HbA_1c_-blood glucose mismatch [3].

## Case Presentation

A 35-year-old woman of Windish ancestry, present-day Slovenia, first presented in 1989 for subacute thyroiditis, which resolved. Her occupational, environmental, drug, and alcohol history were unremarkable. Family history was positive for diabetes mellitus, breast cancer, and pancreatic cancer. She has been routinely evaluated and has no other pertinent medical history. All laboratory results, including enzymatically determined HbA_1c_ and fingerstick blood glucose, remained unremarkable until reexamination in 2013, when HPLC could not determine an HbA_1c_ value due to hemoglobinopathy or other interfering substances. Subsequent HbA_1c_ evaluation via boronate affinity and quantitative capillary electrophoresis (QCE) were conducted with values remaining in the prediabetic range. In 2021, the patient returned for routine evaluation where HPLC yielded an HbA_1c_ greater than or equal to 14.9% (≥139 mmol/mol) and estimated average glucose (EAG) of 381 mg/dL (21.2 mmol/L) (normal reference range, <126 mg/dL to <7.0 mmol/L), despite a fingerstick glucose of 130 mg/dL (7.2 mmol/L) (normal reference range, <126 mg/dL to <7.0 mmol/L).

## Diagnostic Assessment

Physical examination in June 2021 was unremarkable with no reported complaints. Given the elevated HbA_1c_ value without substantial lifestyle changes or symptoms, suspicion of possible interfering substances or hemoglobinopathy was theorized. Repeat HbA_1c_ via QCE revealed an HbA_1c_ of 6.3% (45 mmol/mol), EAG of 134 mg/dL (7.4 mmol/L), and fasting blood glucose of 102 mg/dL (5.7 mmol/L), a notable change from historical values. The discrepancy between EAG and fingerstick glucose warranted further studies to discern the inconsistency, including Hb electrophoresis, capillary electrophoresis, HPLC, mass spectrometry, and isoelectric focusing. Electrophoresis revealed 50.1% HbA (normal reference range, 95%-98%), 2.6% HbA_2_ (normal reference range, 2%), and 47.3% Hb Graz (normal reference range, 0%) of total Hb.

## Treatment

Due to steadily increasing prediabetic fasting glucose and HbA_1c_, metformin was initiated at 500 mg daily in December 2019, with titration to 500 mg 3 times daily by December 2020. Possible theories on the 2021 HbA_1c_ being greater than or equal to 14.9% (≥139 mmol/mol) included alteration of HPLC technology, instrument miscalibration, or the HPLC device not detecting a variant in the sample, allowing the test to be performed. The elution columns vital to identifying Hb moieties within HPLC machines vary based on the manufacturer and may change depending on current technology/laboratory equipment. Further workup was ordered to rule out organic causes. Repeat HbA_1c_ with different methodologies and Hb electrophoresis were performed (Table 1), and the patient was found to be heterozygous for Hb Graz. Repeat HbA_1c_ with QCE provided a value of 6.3% (45 mmol/mol), aligning with the patient's clinical profile.

**Table 1.** Glycated hemoglobin A_1c_ values for a patient with the hemoglobin Graz variant across time

Date, y	Enzymatic (<5.7%) (<39 mmol/mol)	HPLC (<5.7%) (<39 mmol/mol)	Boronate affinity (<5.7%) (<39 mmol/mol)	QCE (<5.7%) (<39 mmol/mol)	CTI (<5.7%) (<39 mmol/mol)	EAG (<126 mg/dL) (<7 mmol/L)	Fasting glucose (126 mg/dL) (<7 mmol/L)
2008	4.3%*^a^* (24 mmol/mol)	N/A	N/A	N/A	N/A	77 mg/dL*^a^* (4.3 mmol/L)	93 mg/dL*^a^* (5.2 mmol/L)
2013	N/A	UTP*^b^*	N/A	N/A	N/A	N/A	N/A
2016	N/A	UTP*^b^*	5.9%*^b^* (41 mmol/mol)	N/A	N/A	123 mg/dL*^b^* (6.8 mmol/L)	110 mg/dL*^b^* (6.1 mmol/L)
2018	N/A	UTP*^b^*	6.0%*^b^* (42 mmol/mol)	6.2%*^b^* (44 mmol/mol)	N/A	130 mg/dL*^b^* (7.2 mmol/L)	96 mg/dL*^b^* (5.3 mmol/L)
2020	N/A	N/A	N/A	6.3%*^b^* (45 mmol/mol)	N/A	134 mg/dL*^b^* (7.4 mmol/L)	121 mg/dL*^b^* (6.7 mmol/L)
2021	N/A	≥14.9%*^b^* (≥139 mmol/mol)	N/A	6.3%*^c^* (45 mmol/mol)	N/A	381 mg/dL*^b^* (21.1 mmol/L)	130 mg/dL*^b^* (7.2 mmol/L)
2023	N/A	N/A	N/A	N/A	4.2%*^b^* (22 mmol/mol)	74 mg/dL*^b^* (4.1 mmol/L)	109 mg/dL*^b^* (6.1 mmol/L)

Methodology used to calculate the HbA_1c_ value is highlighted alongside the fasting glucose taken that same day. Using these values, the EAG was calculated (28.7 × A1c – 46.7 = EAG).

Abbreviations: CTI, colorimetric and turbidimetric immunoinhibition; EAG, estimated average glucose; HbA_1c_, glycated hemoglobin A_1c_; HPLC, high-performance liquid chromatography; N/A, not available; QCE, quantitative capillary electrophoresis; UTP, unable to perform.

^*a*^Lab 1.

^*b*^Lab 2.

^*c*^Lab 3.

## Outcome and Follow-up

The patient continues to have biannual evaluations of her HbA_1c_ values quantified with methods unaffected by Hb Graz. The patient's condition remains stable. She continues metformin 500 mg 3 times per day with no side effects. Given that Hb Graz is an asymptomatic variant, the patient is expected to continue her current therapy with no further diagnostic studies or medication changes.

## Discussion

Hb Graz, first discovered in 1992 in Austria, is an ɑ_2_β_2_2(NA2) histidine to leucine replacement on position 2 of the β-chain of hemoglobin [3]. Its clinical silence enables technical errors to go unnoticed, leading clinicians to misinterpret erroneous HbA_1c_ values as an accurate reflection of the patient's metabolic health. Sources suggest that our case represents the eighth documented patient with Hb Graz worldwide and the first in the United States. Additional reports have documented at least 7 unrelated patients with diabetes and their families in Southern Austria, with potentially more cases, given its asymptomatic nature [4-6].

Due to technological advancements and visits to various laboratories, the patient underwent 5 HbA_1c_ methodologies, including enzymatic analysis, HPLC, boronate affinity, QCE, and colorimetric and turbidimetric immunoinhibition (CTI).

HPLC is the most prevalent methodology used by laboratories to calculate HbA_1c_ at this time due to minimal sample requirement, speed of completion, and precision between samples [7]. HPLC estimates HbA_1c_ based on the charge of Hb moieties. Hb moieties are separated via elution with solvents of increasing strength and then measured by spectrophotometry to calculate the concentration of each Hb moiety [8]. Historically, HPLC struggles to differentiate rare Hb variants from normal Hb, often resulting in erroneously high or low HbA_1c_ values. For example, the Hb Okayama variant in one patient created a falsely elevated HbA_1c_ peak of more than 45% (468 mmol/mol) [9]. A similar discrepancy occurred in this patient, as HPLC resulted in an error message or, on one occasion, an HbA_1c_ of greater than or equal to 14.9% (≥139 mmol/mol). Previous literature on other patients with Hb Graz showed a similar abnormally high HbA_1c_ of 47.4% to 50.4% (495-527 mmol/mol) determined by HPLC [3].

Another methodology, enzymatic analysis, involves the lysis of erythrocytes to free Hb, followed by proteolytic digestion of the glycated N-terminal dipeptide of the β-chain. The resulting glycated residues are oxidized, producing hydrogen peroxide, which reacts with a chromogen to produce a color change measured spectrophotometrically [10]. The enzymatic assay offers reliable HbA_1c_ values even with uncommon Hb variants. However, some rare Hb variants, including Hb Hamilton, Hb G-Copenhagen, and Hb N-Baltimore, demonstrate discrepancies in HbA_1c_ compared to HPLC [9]. For this patient with Hb Graz, enzymatic assay yielded an HbA_1c_ of 4.3% (24 mmol/mol) (see Table 1), which supports its use in this Hb variant subpopulation.

Boronate affinity became the default method for calculating HbA_1c_ when HPLC failed. Boronate affinity chromatography uses columns packed with phenylboronic acid affinity gel variations, sorbitol elutions, and spectrophotometry absorbance studies [11, 12]. Boronate affinity chromatography demonstrates minor interference from rare Hb variants. High levels of HbF may yield falsely low HbA_1c_ values due to the lower glycation rate of this standard Hb variant [11, 13]. For patients undergoing hydroxyurea treatment, HbF will increase, producing artificially low HbA_1c_ values [14]. For our patient, the boronate affinity method produced consistent HbA_1c_ values ranging from 5.9% to 6.0% (41-42 mmol/mol) (see Table 1) across a 2-year time span.

QCE is another method that quantifies Hb fractions based on their electrophoretic and electro-osmotic profiles before quantification by spectrophotometry [15, 16]. In a comparison trial, HbA_1c_ values measured by QCE and HPLC indicated congruence between the 2 methodologies when analyzing HbA_1c_ in Hb variants [17]. Our patient's QCE yielded values of 6.2% to 6.3% (44 to 45 mmol/mol) (see Table 1) across 2 years, whereas HPLC failed to report a value.

CTI is the current method used to evaluate our patient's HbA_1c_. This technique combines colorimetry, which uses spectrophotometry, with turbidimetric immunoinhibition, which measures deposition of antibody-tagged HbA_1c_ turbidimetrically [18]. CTI demonstrates concordance with HPLC in patients with normal Hb variants; however, in rare Hb variants with altered N-terminal β-chains, HPLC yields falsely elevated HbA_1c_ values while CTI remains accurate [19]. However, CTI has limitations, as it relies on antibodies accurately binding HbA_1c_ molecules. This interaction is hindered by thalassemias, hemoglobinopathies, and rheumatoid factors, which alter HbA_1c_ structure or bind the antibody itself (Table 2) [20]. In our patient with Hb Graz, CTI resulted in HbA_1c_ values comparable to enzymatic analysis, supporting its potential use for monitoring glycemic control.

**Table 2.** Common methods of glycated hemoglobin A_1c_ measurement and association with hemoglobin variants

Method	Mechanism	Advantage	Disadvantage	Conditions that potentially affect technique
Enzymatic	Uses enzymes to react with Hb, cleave N-terminal valine, and measure HbA_1c_	Highly specific for HbA_1c_ Technically simple Time-efficient Can be automated Low cost	Susceptible to interference from other substances in blood and Hb variants	Hb Hamilton Hb G-Copenhagen Hb N-Baltimore
HPLC	Separates HbA_1c_ from other hemoglobin forms based on differences in charge and structure	Highly accurate and precise Able to detect most common Hb variants	Expensive Highly technical Time-consuming	Hb Graz Hb Hope Hb S Hb C Hb E Thalassemias
Boronate Affinity	Uses boronate affinity to bind to cis-diol group of glycated Hb	Specific for glycosylated Hb Can also be used to measure other glycated proteins Not affected by Hb variants, beneficial for patients with hemoglobinopathies	Measures all forms of glycated Hb, not just HbA_1c_ Susceptible to interference by other Hb variants	Large amounts of HbF Patients with conditions necessitating treatment with hydroxyurea
QCE	Separates HbA_1c_ from other forms of Hb based on electrical charge differences	High-resolution Can identify Hb variants Requires small sample volume	Requires specialized equipment Requires high technical expertise Affected by Hb variants	HbD-Iran
CTI	Measures color change or turbidity resulting from reaction of HbA_1c_ with specific reagents	Simple Quick Can be automated Low cost	Lower specificity and sensitivity Susceptible to interference from other substances in a sample	Thalassemias Rheumatoid factor autoantibodies

From JM Rhea *Am J Clin Patho,* 2014; 141 (1).

Abbreviations: CTI, colorimetric and turbidimetric immunoinhibition; Hb, hemoglobin; HbA_1c_, glycated hemoglobin A_1c_; HPLC, high-performance liquid chromatography; QCE, quantitative capillary electrophoresis.

The HbA_1c_, EAG, and fasting glucose values depicted in Table 1 highlight the importance of incorporating different methodologies in monitoring patients with hemoglobinopathies. Four of the 5 methods yielded HbA_1c_ with EAG values comparable to the fasting glucose taken that same day. Each methodology has advantages and disadvantages when performing patient personalized testing (see Table 2). Enzymatic, boronate affinity, QCE, and CTI methodologies are typically reliable, even in the setting of hemoglobinopathies. For this reason, such techniques are recommended for patients with a known history or those undergoing a first-time analysis. Compared to the other modalities, HPLC is affected by several rare hemoglobinopathies and can lead to erroneously high values. An inconclusive HPLC test demands further workup and requires a subsequent change in the methodology of HbA_1c_ analysis. Beyond HbA_1c_, clinicians must incorporate alternative glucose monitoring methods for patients in whom HbA_1c_ may be inconclusive. Such methodologies include fructosamine, glycated albumin, and 1,5-anhydroglucitol (1,5-AG) [20]. Fructosamine measures the mean blood glucose concentration over weeks by analyzing the amount of fructose bound to serum protein through glycosylation [20]. The glycated albumin method measures the proportion of serum glycated albumin to the total albumin within the blood. The last alternative method uses 1,5-AG, which remains at a constant concentration during euglycemic states. However, as glucose levels rise, 1,5-AG levels decrease [20]. Researchers have begun to explore the HbA_1c_ values of patients with hemoglobinopathies to create data banks of Hb variants that interfere with HbA_1c_ measurements, thereby aiding clinicians in monitoring these patients [21].

The United States is a heterogeneous population of ethnicities and cultural backgrounds. With this diversity comes the need to acknowledge their differences, especially when considering the implications on their health. The Northern California Comprehensive Thalassemia Center has informative resources on the birth prevalence of varying Hb disorders by race and ethnicity that may be useful when considering hemoglobinopathies [21]. The astute clinician should acknowledge HbA_1c_’s limitations, varying methodologies, and the next steps for values that do not align with patient presentation. If an abnormal value is detected, the method by which the HbA_1c_ was calculated should be verified and compared to prior samples to assess for incongruence. If conflicting data persist, clinicians should consider hemoglobin DNA sequencing to determine the exact hemoglobinopathy present and use past scientific literature to determine the HbA_1c_ methodology best suited for that exact variant. If HbA_1c_ testing continues to produce inconsistent results, clinicians should consider alternative methods for evaluating glucose control. Although many hemoglobinopathies are clinically silent, it is vital for clinicians to understand the most accurate HbA_1c_ methodology to ensure adequate glycemic control of their patients.

## Learning Points

- Multiple methodologies exist to evaluate HbA_1c_, all of which have limitations when evaluating rare hemoglobinopathies. Recognizing when patient presentation and laboratory values are misaligned is essential, indicating the need for further investigation.
- Clinicians should be mindful of the HbA_1c_ methodology employed by their laboratory and possible alternatives for glucose monitoring if the validity of HbA_1c_ values is uncertain.
- Hb Graz is an extremely rare clinically silent Hb variant now documented outside Europe. It can cause confusion due to incongruent HbA_1c_ values across various methodologies.

## Data Availability

Original data generated and analyzed during this study are included in this published article.

## Abbreviations

1,5-AG: 1,5-anhydroglucitol
CTI: colorimetric and turbidimetric immunoinhibition
EAG: estimated average glucose; Hb, hemoglobin
HbA_1c_: glycated hemoglobin A_1c_
HPLC: high-performance liquid chromatography
QCE: quantitative capillary electrophoresis
